# Identification of 9 uterine genes that are regulated during mouse pregnancy and exhibit abnormal levels in the cyclooxygenase-1 knockout mouse

**DOI:** 10.1186/1477-7827-5-28

**Published:** 2007-07-06

**Authors:** Baohui Zhao, Deanna Koon, Allyson L Curtis, Jessica Soper, Kathleen E Bethin

**Affiliations:** 1 Department of Pediatrics and Herman B. Wells Center for Pediatric Research, Indiana University School of Medicine, Riley Hospital for Children, Indianapolis, IN 46202, USA

## Abstract

**Background:**

Preterm birth is the leading cause of all infant mortality. In 2004, 12.5% of all births were preterm. In order to understand preterm labor, we must first understand normal labor. Since many of the myometrial changes that occur during pregnancy are similar in mice and humans and mouse gestation is short, we have studied the uterine genes that change in the mouse during pregnancy. Here, we used microarray analysis to identify uterine genes in the gravid mouse that are differentially regulated in the cyclooxygenase-1 knockout mouse model of delayed parturition.

**Methods:**

Gestational d18.0 uteri (n = 4) were collected from pregnant wild-type and cyclooxygenase-1 knockout mice. Part of the uterus was used for frozen sections and RNA was isolated from the remainder. Microarray analysis was performed at the Indiana University School of Medicine Genomic Core and analyzed using the Microarray Data Portal. Northern analysis was performed to confirm microarray data and the genes localized in the gravid uterus by in situ hybridization.

**Results:**

We identified 277 genes that are abnormally expressed in the gravid d18.0 cyclooxygenase-1 knockout mouse. Nine of these genes are also regulated in the normal murine uterus during the last half of gestation. Many of these genes are involved in the immune response, consistent with an important role of the immune system in parturition. Expression of 4 of these genes; arginase I, IgJ, Tnfrsf9 and troponin; was confirmed by Northern analysis to be mis-regulated during pregnancy in the knockout mouse. In situ hybridization of these genes demonstrated a similar location in the gravid wild-type and Cox-1 knockout mouse uteri.

**Conclusion:**

To our knowledge, this is the first work to demonstrate the uterine location of these 4 genes in the mouse during late pregnancy. There are several putative transcription factor binding sites that are shared by many of the 9 genes identified here including; estrogen and progesterone response elements and Ets binding sites. In summary, this work identifies 9 uterine murine genes that may play a role in parturition. The function of these genes is consistent with an important role of the immune system in parturition.

## Background

In 2004 12.5% of all births in the USA were preterm [1]. Preterm birth is the leading cause of all infant mortality and a major cause of morbidity [2-4]. The reason that idiopathic preterm labor remains an enigma is that the mechanisms that initiate normal labor are largely unknown. Parturition has been studied in many species, but there is no perfect animal model of human labor [5]. Mouse models are useful to study parturition because gestation is short (19.5 days) and genetically modified models are readily available [6]. In addition, some of the uterine changes that occur in pregnancy are similar in mice and humans [6-8].

Gene knockouts of prostaglandin synthesis enzymes and receptors have demonstrated the importance of the prostaglandin synthesis pathway for normal murine parturition [9-12]. Cyclooxygenase-1 (Cox-1) and Cox-2 catalyze the first committed step in prostaglandin synthesis. Cox-1 and Cox-2 have similar structure and are both inhibited by the nonselective nonsteroidal antiinflammatory drugs, but are regulated differently [13]. Cox-1 is constitutively expressed in most tissues. However, induction of uterine Cox-1 mRNA between gestational days (d) 14.5 and 15.5 and the subsequent increase in prostaglandin F2α (PGF2α) are critical to normal timed labor in the mouse [9,10]. In murine parturition, arachidonic acid is released from cell membranes by cytosolic phospholipase A2 (cPLA_2_). Cyclooxygenase-1 (Cox-1) and prostaglandin F synthase convert arachidonic acid to PGF2α which causes luteolysis and a fall in progesterone resulting in induction of uterine prostaglandin receptors, oxytocin receptor and connexin-43, leading to increased contractions and pup delivery [13]. In normal parturition, progesterone falls between d17.5 and d18.5, followed by an increase between d18.5 and d19.3 of myometrial oxytocin receptor, PGF2α receptor and connexin-43 [10,14,15]. Fertility in the Cox-1 knockout (KO) mouse is normal, but the mice deliver their pups 2 days late. Failure of induction of Cox-1 and PGF2α results in delayed luteolysis, high progesterone levels, decreased levels of oxytocin receptor and connexin-43 (Muglia, unpublished results) on d19.0 (half-day prior to normal delivery) [10,16,17]. Cox-2 is undetectable in most tissues, but can be induced to high levels in response to inflammatory stimuli. The Cox-2 KO mouse is infertile due to defects in ovulation, fertility, implantation and decidualization [18]. Just before delivery, Cox-2 is induced in the myometrium, but does not appear to be important for normal timing of parturition [19-21].

The cPLA_2 _and PGF2α receptor KO mice also fail to deliver their pups normally and demonstrate failure to induce prostaglandin receptors, oxytocin receptor and connexin 43 [10,11,16,19,20]. Bilateral ovariectomy or administration of a progesterone antagonist (RU-486) induces uterine oxytocin receptor and connexin-43 and leads to pup delivery within 16–24 hours in the Cox-1, cPLA_2 _and PGF2α receptor KO mice. Administration of PGF2α also induces delivery in the Cox-1 and cPLA_2 _KO mice [10,11,16,19,21-23]. Since some parturition-related genes have low levels in these murine models that deliver their pups late but are increased to normal after labor is induced, we hypothesized that the Cox-1 KO mouse model of delayed parturition will be useful in identifying other genes important in the parturition pathway.

Microarray analysis identifies hundreds of uterine genes that change during pregnancy [24-26]. Using a cut-off of 2-fold, 504 uterine genes were identified by microarray analysis that are up- or down-regulated on gestational d16.5 or d19.0 compared to d13.5 in the gravid wild-type mouse [24]. To reduce this list of potential parturition-related genes, we compared the uterine gene expression profiles of the gravid d18.0 Cox-1 KO and wild-type mice. By identifying uterine genes that are regulated in normal pregnancy and abnormally expressed in the Cox-1 KO model of delayed parturition, we hypothesized that the number of candidate genes for roles in parturition would be reduced.

In normal mouse pregnancy, but not in the Cox-1 KO mouse, increased uterine PGF2α leads to luteolysis followed by a fall in progesterone between d17.5 and d18.5 [10,15]. For this study, we wished to identify uterine genes activated or repressed by the parturition cascade, independent of progesterone-regulation. Therefore, we harvested uteri from pregnant mice on gestational d18.0.

In summary, we have identified 277 uterine genes that are abnormally expressed in the gravid d18.0 Cox-1 KO mouse. From these genes we have identified 9 uterine genes that are also regulated during normal murine pregnancy and thus may play a role in normal parturition.

## Methods

### Animal husbandry

All mouse protocols were in accordance with National Institutes of Health guidelines and approved by the Institutional Animal Care and Use Committee of Indiana University School of Medicine (Indianapolis, IN). All mice used were 8–16 wks old and of a C57BL/6 (wild-type) or C57BL/6 × 129P2 (Cox-1 KO) genetic background. Mice were housed on a 12-h light, 12-h dark cycle with ad libitum access to rodent chow and water. Mating of estrous females to stud males was confirmed via detection of a copulation plug, with the morning of plug detection designated as d0.5 of gestation. Plugged females were removed from the male cage to ensure accurate gestation timing. Non-gravid (n = 2), gravid gestational d13.5, d16.5, d18.0 and d19.0 wild-type (n = 3–4) and Cox-1 KO mice (n = 3–4) as well as gestational d20.0 and d21.0 Cox-1 KO mice were euthanized by CO_2 _inhalation, and then the uteri were quickly isolated. The fetuses, fetal membranes and placenta were removed. Three pieces of uterus from each mouse were fixed in 4% paraformaldehyde, dehydrated in 10% sucrose and embedded in OCT (Sakura Finetek, Torrance, CA), for sectioning on a cryostat. The remainder of the uterus was snap frozen in liquid nitrogen and kept at -80°C until RNA was isolated.

### RNA isolation and microarray analysis

Gestational d18.0 RNA (n = 4 for each genotype) was isolated using Trizol (Invitrogen, Carlsbad, CA) and further purified using an RNeasy Mini Kit (Qiagen, Valencia, CA) and dissolved in Rnase-free water. RNA from all other time points was isolated using Trizol only. The A_260_/A_280 _was 1.55 – 1.69 for all Rneasy purified samples. The A_260_/A_280 _for Trizol-purified RNA was 1.43–1.6. Integrity of RNA was further checked on a 1.2% formaldehyde agarose gel. Concentration of RNA was determined by the A_260 _and amounts adjusted according to the relative intensities of the 28S band on the test gel. Approximately 200–300 μg of RNA was isolated from each uterus. The Center for Medical Genomics Core at Indiana University School of Medicine analyzed the RNA using Affymetrix 430 2.0 GeneChips. Five μg of total RNA was labeled following single cycle protocols recommended in the GeneChip Expression Analysis Technical Manual (Affymetrix, Santa Clara, CA). The cDNA was synthesized from RNA using a T7 promoter-dT24 oligonucleotide primer with the Invitrogen Life Technologies SuperScript Choice system. After the second strand synthesis and incubation with T4 DNA polymerase the products were purified using an Affymetrix Cleanup Module. Biotynylated cRNA was made using the Affymetrix IVT kit. The cRNA was purified with Qiagen RNeasy columns, quantitated and then fragmented at high temperature with magnesium. Fifteen μg of biotinylated cRNA was added to a total hybridization cocktail of 300 μL and 200 μL was hybridized to a 430 2.0 GeneChip at 45° for 17 h with constant rotation. The GeneChips were washed, stained with phycoerythrin-labeled streptavidin, washed, incubated with biotinylated anti-streptavidin and then restained with phycoerythrin-lableled streptavidin to amplify the signals, all following the standard Affymetrix protocol. In order to reduce non-random error, balanced groups of samples were handled in parallel. GeneChips were then scanned using a dedicated scanner controlled by Affymetrix GCOS software. The images were examined for defects and the hybridization intensity data was analyzed with the Affymetrix Microarray Suite version 5 (MAS5). MAS5 calculated a set of metrics that describe probe performance. The average intensity on each array was normalized by global scaling to a target intensity of 1000. Data were imported into the MicroArray Data Portal for further analysis. All genes included in the analysis had to have a "present" call in at least 50% of the samples. Significance was determined by a Student's t test of the log-transformed signal with a Welch's correction. Gene changes were considered significant if the p value was less than or equal to 0.05 and the fold-change was at least 1.5. Expression levels of all genes on the GeneChips are available under GEO accession number GSE8269 [27].

### *In situ *hybridization

The paraformaldehyde fixed uteri were cryopreserved in 10% sucrose in PBS and then embedded in OCT compound (Sakura Finetek, Torrance, CA) for sectioning on a cryostat. Sixteen-micrometer cryostat sections through the uteri were collected and thaw-mounted onto Superfrost plus slides. The slides were vacuum dried overnight and stored at -80°C [28]. RNA probes were prepared with α-^33^P-uridine triphosphate (Perkin Elmer, NJ) by incubating 2 μg of the linearized template with the appropriate polymerase using the Promega Riboprobe System [29]. RNA probes for arginase I [GenBank: NM_007482] and IgJ [GenBank: NM_152839] were transcribed from mouse EST clones (ATCC, Manassas, VA) using T7 polymerase. For Tnfrsf9 [GenBank: NM_011612] and Tnni2 [GenBank: NM_009405] RNA from gravid d13.5 normal mouse uteri was reverse transcribed and then amplified using specific primers designed by Oligoperfect (Invitrogen, Carlsbad, CA) (Table 1). The PCR products were TOPO cloned (Invitrogen, Carlsbad, CA) following the manufacturer's instructions. DNA was isolated from the bacterial culture using Qiagen Maxi kit (Valencia, CA). Both Tnfrsf9 and Tnni2 were linearized with EcoRI. Unincorporated nucleotides were removed by purifying the riboprobe over a Sephadex G50 column (Roche). Probes were used at a concentration of 1 × 10^6 ^cpm/mL of the radiolabel. Slides were hybridized to the appropriate anti-sense riboprobes in a humidified chamber for 20 h at 62°C for IgJ and 60°C for all other probes as described [28]. After washes, with the most stringent wash in 0.1 × SSC at 65°C for 30 min, and vacuum drying, slides were exposed to BioMax MR film (Kodak, Rochester, NY) for 1–2 days. Slides were dipped in NBT Autoradiography emulsion (Kodak) and exposed for 4 days (Tnfrsf9) or 8 days (arginase, IgJ and Tnni2). Emulsion dipped slides were developed and fixed using D-19 Developer and Fixer (Kodak). Slides were counterstained with hematoxylin and eosin. Sense probes were generated from the same EST clones used for the antisense probes. Sense templates were linearized with Not I. Arginase and IgJ sense templates were transcribed with SP6 polymerase. Tnfrsf9 and Tnni2 sense templates were transcribed with T7 polymerase. The sense probes were incubated under the identical conditions and the emulsion was exposed for the identical length of time as the corresponding antisense probes. Four mice from each gravid time point were analyzed for *in situ *hybridization. Since we were focusing on gravid uterus, only 2 wild-type uteri were analyzed. A representative slide from each time point is shown in the figures.

**Table 1:** Primers used to amplify Tnfrsf 9 and Tnni2 from gravid d13.5 mouse uterus.

Gene	Direction	Sequence	Exons
Tnfrsf9	Forward	5'CTAGTGGGCTGTGAGAAGGT3'	3–9
	Reverse	5'TCCTCCTTCTTCTTCCTGTG3'	
Tnni2	Forward	5'GGAGACAGCACCTGAAGAGT3'	3–6
	Reverse	5'CCGTTCCTTCTCAGTGTCTT3'	

### Northern blots

Northern analysis was performed as described previously [28]. Ten μg of RNA was separated on a 1.2% formaldehyde agarose gel and transferred overnight to nitrocellulose membranes. RNA on the blots were fixed by vacuum drying at 80°C (2 hours). RNA probes specific for mouse arginase I, IgJ, Tnfrsf 9, Tnni2 or cyclophilin A (as a loading control) (Ambion, Austin, TX) labeled with α-[^32^P]UTP (Perkin Elmer, Wellesley, MA) were generated as described for the *in situ *probes above. The blots were hybridized with the appropriate probe at 65°C overnight in 50% formamide-containing buffer. After being washed, the blots were used to expose Biomax-MS film (Kodak). Each mRNA hybridization signal was corrected for loading and recovery by normalization to the intensity of cyclophilin A hybridization. The films were scanned into Photoshop using an Epson Perfection 3490 Photo scanner. The band intensities were quantitated using NIH Image J [30]. A two-tailed ANOVA was used to determine statistical significance. For d18.0 samples 5 mice of each genotype were used. Only 3 mice from all other time points were used to be able to fit all of the Cox-1 KO samples on a single gel. The wild-type and Cox-1 KO blots were incubated at the exact same time in the same cylinder with radiolabeled probe so that relative expression levels could be compared.

### Transcription factor binding sites

Putative binding sites for each gene were determined using the VISTA browser and rVISTA program [31-34]. Only putative transcription factor binding sites that were conserved in both the mouse and human genes were identified.

## Results and discussion

Despite advances in medical technology, the incidence of preterm birth continues to increase and is the leading cause of infant mortality. We have had only limited success in preventing preterm labor because normal labor is not completely understood. We have studied parturition in the mouse because gestation is short and genetic manipulation of the mouse is relatively easy. We hypothesized that uterine genes that are abnormally expressed in a mouse model of delayed parturition may play roles in normal parturition and be good targets for therapeutic prevention of preterm labor. Since the Cox-1 KO mouse delivers its pups 2 days late, we compared uterine gene expression from the gravid d18.0 Cox-1 KO and wild-type mice. In order to reduce the number of candidate parturition-related genes identified we compared the genes identified as abnormally expressed in the Cox-1 KO uterus to the genes that we had previously identified as changing from gestational d13.5 to d19.0 in the wild-type mouse uterus [24]. Microarray analysis was performed on gravid d18.0 wild-type (n = 4) and Cox-1 KO (n = 4) mouse uterine RNA. We detected 277 genes that were either up- or down-regulated at least 1.5 fold in the Cox-1 KO uterus (p < 0.05) (See additional file 1: Excel table 1 for all 277 genes identified.). The majority of these genes did not change significantly between d13.5 and d19.0 in the gravid wild-type uterus. However, 9 of these genes are regulated in the wild-type gravid mouse uterus after the induction of Cox-1 (d19.0 compared to d13.5) (Tables 2 and 3). The function of several of the 9 identified genes involves the immune system. Other functions include proteolysis, contraction regulation and a regulator of nitric oxide synthase.

**Table 2:** Uterine genes regulated in normal mouse pregnancy and up-regulated in the gravid d18.0 Cox-1 KO mouse

Gene Symbol	Genbank	Cox-1 KO vs WT^1^	Fold (d19/d13.5)^a^
IgJ	NM_152839	5.8	3.1
Arg1	NM_007482	1.8	4.6
Gzme	NM_010373	1.5	-10.0
Tnfrsf9	NM_011612	1.5	-2.5

^1^COX-1 KO (knockout) mouse compared to WT (wild-type) by microarray analysis. ^a^Bethin et al. 2003; fold difference in wild-type gravid mouse uterus on d19.0 compared to d13.5 by microarray. p values are all < 0.05

**Table 3:** Uterine genes regulated in normal mouse pregnancy and down-regulated in the gravid d18.0 Cox-1 KO mouse

Gene Symbol	Genbank	Cox-1 KO vs WT^1^	Fold (d19/d13.5)^a^
Klk6	NM_010639	-1.5	-2.5
Gem	NM_010276	-1.5	-2.0
Cftr	NM_021050	-1.5	2.5
Atp8a1	NM_009727	-1.6	11.0
Tnni2	NM_009405	-1.6	-5.0

^1^COX-1 KO (knockout) mouse compared to WT (wild-type) by microarray analysis. ^a^Bethin et al. 2003; fold difference in wild-type gravid mouse uterus on d19.0 compared to d13.5 by microarray. p values are all < 0.05

Gestational d18.0 was chosen to obtain the latest point in gestation before effects from differences in progesterone levels in the Cox-1 KO and wild-type mice would affect gene expression. To confirm that progesterone differences did not affect expression of the identified genes, we compared expression of 16 known progesterone-regulated uterine genes in the wild-type and knockout mice (Table 4) [35-38]. Kallikrein 6 (Klk6), which is up-regulated in response to acute progesterone exposure, was decreased in the Cox-1 KO mice. However, none of the other 15 known progesterone-regulated genes showed a significant difference in expression levels in the Cox-1 KO compared to wild-type mice (Table 4). This is consistent with the differences in uterine gene expression seen in the Cox-1 KO mice on d18.0 being independent of progesterone regulation.

**Table 4:** Fold-change in the gravid d18.0 Cox-1 KO mouse uterus known progesterone-regulated genes compared to wild-type

Gene Symbol	Cox-1 KO vs. WT	GenBank
Ihh	1.09	NM_010544
Fst	1.01	NM_008046
Areg	signal too low	NM_009704
Hdc	1.08	NM_008230
Hoxa10	-1.09	NM_008263
Hoxa11	-1.12	NM_010450
IGF-1	-1.36	NM_184052
Irg1	-1.24	L38281
PR	-1.04	NM_008829
Calca	1.2	NM_007587
Klk6	-1.48*	NM_010639
MMP11	1.2	NM_008606
CSPG2	1.1	NM_001081249
Col6A3	-1.02	NM009935
Cdh11	-1.25	NM_009866
Cst3	1.03	NM_009976

^1^Expression by microarray analysis in the Cox-1 KO (knockout) compared to WT (wild-type) mice.

* p < 0.05

**Figure 1:**
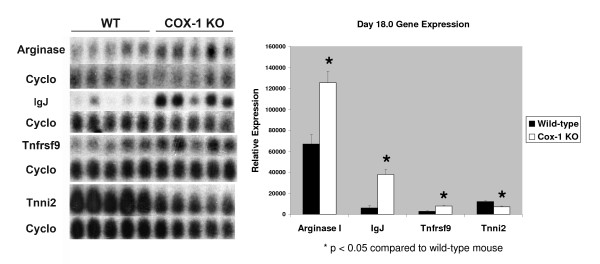
**Blot and graph of Northern analysis of arginase, immunoglobulin J chain, Tnfrsf9 and troponin levels from gravid d18.0 wild-type (WT) and Cox-1 KO uteri (n = 5)**. Error bars show the standard error.

**Figure 2:**
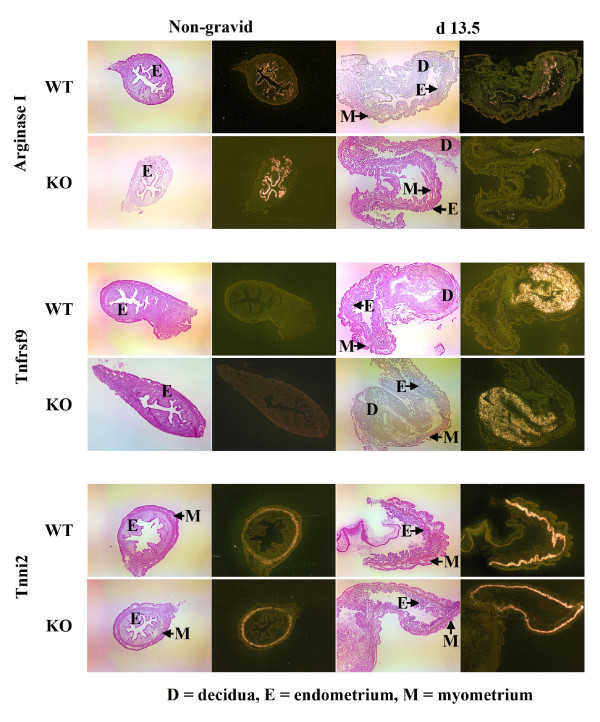
***In situ *hybridization for arginase, Tnfrsf9, and Tnni2 in the non-gravid and gravid d13.5 wild-type and Cox-1 KO mouse uteri**. *In situ *hybridization was performed on (n = 4) each gravid time point and (n = 2) non-gravid mice. Shown are representative light-field images (left) and dark-field images (right) from non-gravid (NG) (50×) or gravid d13.5 uteri (25×). The slides were counterstained with hematoxylin and eosin. Areas of hybridization appear white, demonstrating deposition of silver granules.

**Figure 3:**
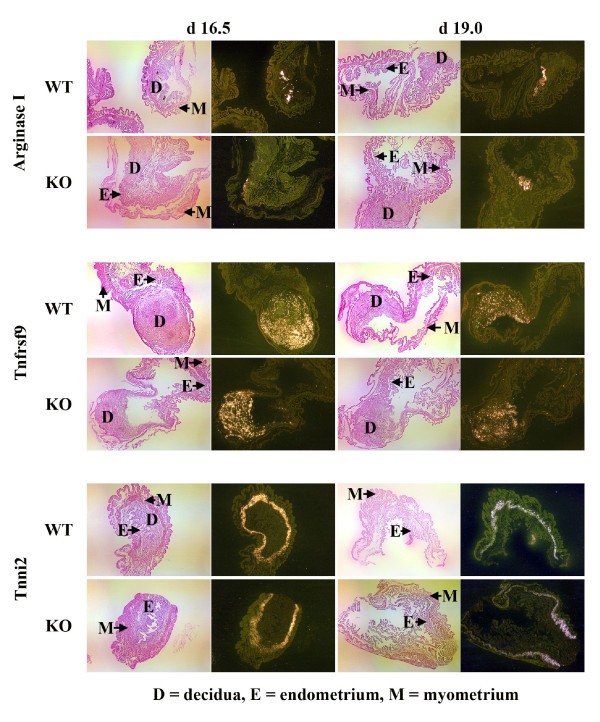
***In situ *hybridization for arginase, Tnfrsf9, and Tnni2 in the gravid d16.5 and d19.0 wild-type and Cox-1 KO mouse uteri**. *In situ *hybridization was performed (n = 4) from each gravid time point. Shown are representative light-field images (left) and dark-field images (right) from gravid d16.5 and d19.0 uteri (25×). The slides were counterstained with hematoxylin and eosin. Areas of hybridization appear white, demonstrating deposition of silver granules.

**Figure 4:**
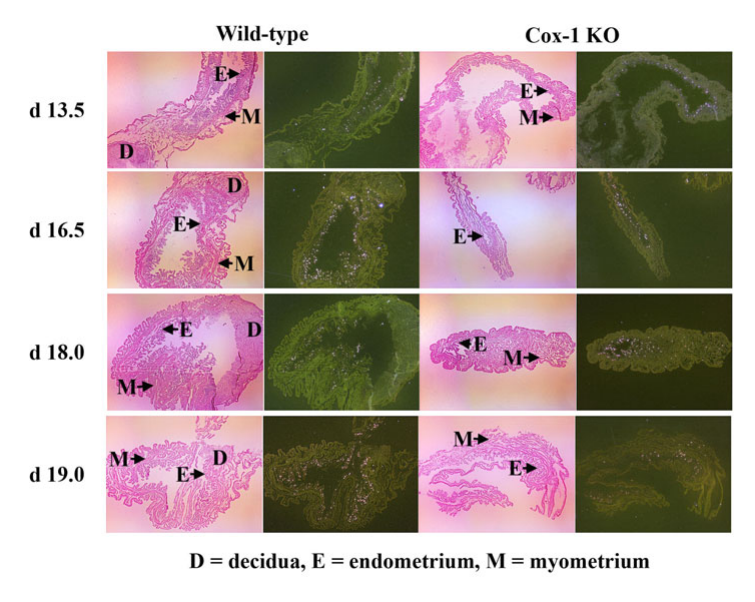
***In situ *hybridization for IgJ in the gravid d13.5, d16.5, d18.0 and d19.0 wild-type and Cox-1 KO mouse uteri**. *In situ *hybridization was performed (n = 4) from each gravid time point and non-gravid mice (n = 2). Shown are representative light-field images (left) and dark-field images (right) (25×). The slides were counterstained with hematoxylin and eosin. Areas of hybridization appear white, demonstrating deposition of silver granules.

**Figure 5:**
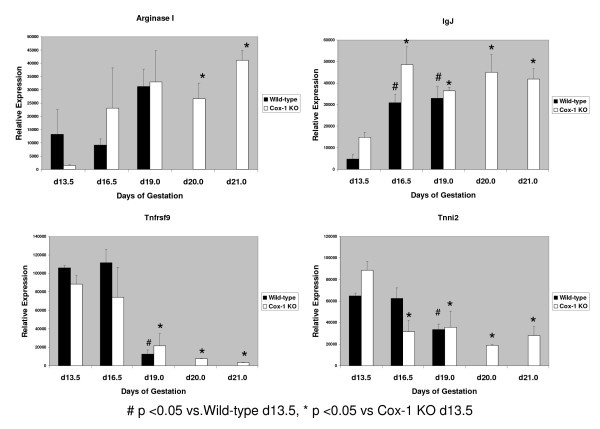
**Bar graphs for Northern analysis of arginase, immunoglobulin J chain, Tnfrsf9 and troponin**. Ten μg of RNA isolated from gravid d13.5, d16.5 and d19.0 wild-type (n = 3) and d13.5, d16.5, d19.0, d20.0 and d21.0 Cox-1 KO (n = 3) uteri were run on the gel. Error bars show the standard error.

From the 9 candidate genes we chose the 3 genes that were up-regulated the most and one of the genes that was down-regulated the most in the Cox-1 KO mice compared to the wild-type mice to study further by Northern analysis: arginase I (liver type), immunoglobulin J chain (IgJ), tumor necrosis factor receptor superfamily 9 (Tnfrsf9) and troponin I (Tnni2) (skeletal, fast) (Fig. 1). *In situ *hybridization showed that all 4 genes were expressed in the same location and distribution in the Cox-1 KO mouse and the wild-type mice (Figs. 2, 3, 4). There was no hybridization signal detected for any of the sense probes for these genes (data not shown). Northern analysis was also done on gravid uterus from wild-type d13.5, d16.5 and d19.0 (n = 3) and Cox-1 KO d13.5, d16.5, d19.0, d20.0 and d21.0 (n = 3) mice (Fig. 5). The results were consistent with the relative expression levels demonstrated on the microarray analysis for the wild-type mice. Northern analysis was not done on the remaining genes. However, *in situ *hybridization was performed on Klk6, Gem, Cftr and Atp8a1 (data not shown) and the signal intensity qualitatively agreed with the microarray data. *In situ *hybridization of arginase, Tnfrsf9 and Tnni2 d18.0 looked similar to d19.0 in situs (data not shown).

Arginase I (AI) expression on d18.0 was 1.9- and 1.8- fold greater in the Cox-1 KO uterus (p = 0.004), by Northern and microarray analysis, respectively (Fig. 1, Table 2). Expression of AI is limited to a small part of the endometrium in the gravid uterus (Figs. 2 &3). By microarray analysis AI expression in the gravid wild-type uterus is increased 4.6-fold on d19.0 compared to d13.5 [24]. By Northern analysis, there is a trend for AI expression to increase on d19.0 compared to d13.5 in both the wild-type and Cox-1 KO mice but this did not meet statistical significance (Fig. 5). AI is primarily expressed in the liver but has been found in other cells, including the endometrium [39-43]. AI catalyzes the hydrolysis of arginine to urea and ornithine thus inhibiting nitric oxide synthase as well as controlling DNA, RNA and protein synthesis [44]. Pregnancy or administration of estradiol increases uterine arginase activity [45,46]. AI RNA has been shown to be present in the non-gravid mouse endometrium, but to our knowledge has not been evaluated in mouse pregnancy [39]. In the pregnant mouse, AI may be both providing substrate for growth of the fetus and placenta and promoting uterine contractions by inhibition of nitric oxide synthase [45-50]. Our data are consistent with a factor other than Cox-1 produced prostaglandins inducing AI in the uterus. Inhibition of Cox-2 but not Cox-1 has been shown to reduce arginase activity [51]. Increased T helper_2 _(Th_2_) cytokines in the Cox-1 KO mouse may be responsible for the higher levels of AI in the Cox-1 KO mouse [52]. Induction of AI at the end of pregnancy in both wild-type and knockout mice could be augmenting uterine contractions via inhibition of nitric oxide synthase. Increased arginase I in the Cox-1 KO may compensate for failure of the normal parturition genes to be activated allowing nitric oxide synthase to be inhibited enough that the Cox-1 KO mice finally deliver their pups.

Immunoglobulin J chain (IgJ) is expressed late in B cell maturation and is important for the primary immune response [53]. IgJ is present in non-pregnant human endometrium, up-regulated in laboring human myometrium and is present in fetal membranes and decidua during the first trimester of pregnancy [26]. By microarray analysis, IgJ is up-regulated 2.7-fold between gestational d13.5 and d16.5 in the pregnant wild-type mouse suggesting that the factor regulating Cox-1 induction may also be regulating B cell and IgJ induction [24]. By Northern analysis IgJ expression increases in both the wild-type and Cox-1 KO mice between d13.5 and d16.5 and is higher in the gravid Cox-1 KO mouse at all time points (Fig 5). On d18.0, Immunoglobulin J (IgJ) expression is increased 6.2-fold (p = 0.0003) by Northern and 5.8-fold by microarray in the Cox-1 KO mouse (Fig. 1, Table 2). IgJ is expressed in distinct cells in the endometrium (Fig. 4). These data are consistent with increased Th_2 _cytokines in the Cox-1 KO mouse stimulating B cell proliferation in the gravid uterus, resulting in increased IgJ expression. IgJ is likely playing a role in parturition involving immune system activation near the end of pregnancy. Its expression appears to be related to a parturition signal that is simultaneous with or earlier than the induction of Cox-1.

Tumor necrosis factor receptor superfamily 9 (Tnfrsf9, 4-1BB, CD137) is expressed in immune cells, including placental macrophages, and plays an important role in the regulation of the immune response [54]. Activation of Tnfrsf9 induces proliferation of both CD8^+ ^and CD4^+ ^T cells and has been shown to induce rejection of tumors, enhance T cell response to viruses, reduce autoimmune disease progression and suppresses Th_2 _cells [54,55]. Tnfrsf9 uterine expression on d18.0 is 2.7- and 1.5- fold higher (p = 0.0003) in the Cox-1 KO uterus than the wild-type mouse by Northern and microarray analysis, respectively (Fig. 1, Table 2). By microarray analysis Tnfrsf9 mRNA is down-regulated 2-fold between d13.5 and d19.0 in the gravid wild-type uterus [24]. This was confirmed by Northern analysis, which also demonstrated a decrease between d13.5 and d19.0 in the gravid Cox-1 KO (Fig. 5). In the pregnant mouse, uterine Tnfrsf9 is present in the implantation site (Figs 2 &3). The role of down-regulation of Tnfrsf9 in pregnancy may be to induce Th_2 _cells which then induce arginase and IgJ. Our data suggest that prostaglandins may suppress Tnfrsf9 but are not the major regulator since Tnfrsf9 decreases in the Cox-1 KO mice as well.

Troponin I type 2 (Tnni2) inhibits the actomyosin ATPase and provides a calcium-sensitive switch for muscle contraction and also directly inhibits calcium flux by binding the ryanodine receptor [56-58]. On d18.0 expression of Tnni2 is decreased in the Cox-1 KO to 1.6-fold of the wild-type mice (p = 0.0005) by Northern and microarray analysis (Fig. 1, Table 3). Tnni2 is expressed in the circular layer of the myometrium (Figs. 2 &3). To our knowledge, this is the first report of Tnni2 in the gravid uterus. Microarray analysis shows that Tnni2 expression decreases 5.9-fold between d13.5 and d19.0 in the gravid wild-type mouse uterus. Northern analysis confirms these data and demonstrates a decrease in Tnni2 expression earlier in the Cox-1 KO on d16.5 (Fig. 5). Tnni2 in the gravid uterus may be helping to maintain the uterus in a quiescent state both directly by inhibiting actomyosin and indirectly by inhibiting calcium influx. In prepubertal rats, high dose estrogen down-regulates uterine Tnni2 [59]. In rodent pregnancy, estradiol increases just prior to delivery [6,60]. In wild-type mice, the fall in Tnni2 appears to correspond with the normal increase in estrogen just prior to delivery. The earlier decrease of Tnni2 in the Cox-1 KO mouse suggests that another factor also regulates Tnni2.

In an effort to find similarities among the 9 genes that have abnormal expression levels in the Cox-1 KO uterus and are regulated after d13.5 in the wild-type mouse, the promoter regions of each gene and Cox-1 were evaluated with rVISTA. Seven of the genes contain at least 1 estrogen response element and at least 1 putative Ets binding site. In addition, 6 genes contain a putative Gli binding site and 5 genes contain a putative progesterone response element (Table 5). Gli3 is a zinc finger transcription factor that is a downstream mediator of the sonic hedgehog pathway [61]. It is expressed from early in development through adulthood. It has been described in both the human and murine uterus and may play a role in implantation [62,63]. Elf3 is a member of the Ets family of transcription factors whose expression is limited to the epithelium. Elf3 is important for development of the intestine, but its role in the uterus is unknown [64]. By microarray analysis of gravid wild-type murine uterus Elf3 and Ets2 are increased on d19.0, 5.0-and 1.7-fold, respectively, compared to d13.5 [24]. On d18.0 we did not see a difference between the Cox-1 KO and wild-type mice in either of these transcription factors. Therefore, Elf3 and Ets2 could only be modifying transcription of parturition genes on d18.0 if estrogen and/or PGF2α modify the binding of these transcription factors. Gli3 levels on d19.0 decrease to 18% of d13.5 in the gravid wild-type mouse, but no differences in Gli3 levels were detected in the Cox-1 KO mouse [24]. Estradiol increases and progesterone decreases near term in the pregnant mouse [6,60]. Since progesterone-regulated genes were unaffected in the d18.0 Cox-1 KO mouse, estrogen is likely playing a larger role than progesterone in these gene changes.

**Table 5:** Conserved putative transcription factor binding sites in the mouse and human sequences of the genes identified

Gene Symbol	ERE^1^	ETS	GLI	PRE^2^
IgJ	Yes	Yes		Yes
Coro1a	Yes	Yes	Yes	Yes
Arg1	Yes	Yes	Yes	
Gzme				
Tnfrsf9	Yes			
Klk6				
Gem	Yes	Yes	Yes	Yes
Cftr	Yes	Yes	Yes	Yes
Atp8a1	Yes	Yes	Yes	Yes
Tnni2	Yes	Yes	Yes	Yes
Cox-1	Yes	Yes	Yes	

^1^ERE = estrogen response element, ^2^PRE = progesterone response element

There are numerous differences between murine and human pregnancy. The maintainence of the corpus luteum in mice maintains pregnancy but has no role after the first trimester in humans. Although Cox-1 is critical for normal labor in the mice, it is the Cox-2 isoform that appears to be important in human labor. However, prostaglandins play a key role in myometrial contractions in both mice and humans and studies in knockout mice continues to be useful in elucidating the hormonal milieau of pregnancy [9].

## Conclusion

Although care of the infant born too soon has dramatically improved over the last 40 years, the incidence of preterm births continues to rise [65]. One of the major reasons that we have had only limited success in treating preterm labor is that we do not fully understand normal labor. Up to 50% of preterm births can be attributed to infection [66]. However, idiopathic preterm labor continues to represent a large percentage of the preterm births. There are data that implicate genetics in the timing of labor, including preterm and postterm delivery [67-70]. We hypothesized that a mouse model of delayed parturition would help us identify genes that are important in normal labor and thus provide possible targets for treating preterm labor. This work identified 9 uterine genes that are regulated during murine pregnancy and demonstrate abnormal levels in the gestational d18.0 Cox-1 KO mouse. We have confirmed these data by Northern analysis for 4 genes: arginase I, IgJ, Tnfrsf9 and Tnni2. We also demonstrated the location of these genes in the gravid mouse uterus. To our knowledge, this is the first time these genes have been investigated and localized in the late pregnant mouse uterus. Of the 4 genes studied, Tnfrsf9, IgJ and arginase I play a role in the immune system. These data are consistent with an important role for the immune system, especially Th_2 _cells, in parturition. These data are consistent with the works of others implicating immune modulators in both pregnancy maintenance and parturition [65]. These data also suggest that studying immune system modifiers that affect Th_2 _cells, specific inhibitors of arginase I and the effects of arginine on uterine contractility may help identify new drugs for the treatment of preterm labor. Drugs that increase expression of Tnni2, which we identified as decreasing at the end of normal pregnancy, may help maintain uterine quiescence and are another promising area of research related to preterm labor. Since many of the genes identified in this work have putative binding sites for Gli3 and Elf3, 2 transcription factors that are up- or down-regulated in the uterus of pregnant wild-type mice, targeting these transcription factors may also affect the timing of labor. Interestingly, despite a deficiency in Cox-1 derived prostaglandins in the Cox-1 KO mouse, none of the gene changes in this delayed model of parturition appear to be the result of decreased prostaglandins.

## Competing interests

The author(s) declare that they have no competing interests.

## Authors' contributions

BZ performed in situ hybridizations, statistical analysis and participated in the design of the study and Northern analysis. DK, AC and JS participated in the care of the mice, timed pregnancies, tissue harvest and RNA isolation and participated in the Northern analysis. KB participated in the design of the study and drafted the manuscript. All authors read and approved the final manuscript.

## Supplementary Material

Additional file 1 **Microarray results of differentially expressed genes in the Cox-1 KO gravid d18.0 mouse uterus Microsoft Excel**. Gravid d18.0 wild-type (n = 4) and Cox-1 KO (n = 4) murine uterus was harvested and RNA isolated. Microarray analysis was done on each sample using Affymetrix 430 2.0. Data were analyzed using Microarray Data Portal. Only genes that were 1.5-fold or greater different between the wild-type and knockout mice with a p < 0.05 are shown. Click here for file
